# Fifty years of continuous hemodialysis: a case of exceptional longevity in a male patient with Alport syndrome

**DOI:** 10.1093/ckj/sfag253

**Published:** 2026-08-05

**Authors:** Stefan Becker, Davor Marinac, Partha Das, Christiane Erley

**Affiliations:** MVZ DaVita, Duisburg, Germany; MVZ DaVita, Duisburg, Germany; DaVita International, London, UK; DaVita Deutschland AG, Hamburg, Germany

To the Editor,

Hemodialysis (HD) has transformed the management of end-stage kidney disease since the 1960s, yet survival for several decades remains exceptional. We report a 70-year-old male with Alport syndrome who has received thrice-weekly HD continuously for 50 years, beginning in 1975, illustrating the boundaries of long-term dialysis survival.

The patient was diagnosed with Alport syndrome based on a family history consistent with hereditary nephritis; molecular diagnostics were unavailable at the time. He commenced chronic HD on 1 May 1975, when dialysis capacity in Germany was severely limited. A Cimino arteriovenous fistula, created on 15 March 1975 in the left forearm, remains fully patent to date without surgical revision, angioplasty, or regular ultrasound surveillance. For the first 8 years, his father performed home HD using a Fresenius 2008 system. Dialysis initially used a Nephral 400 dialyzer with low-potassium (2 mmol/l) and 1.5 mmol/l calcium dialysate; he has since transitioned to high-flux membranes, most recently a Fresenius FX 80. Between 1983 and 2011, he underwent standard in-center HD (three sessions weekly, 5 hours each) before transitioning to extended nocturnal HD from 2011 to June 2024, and back to daytime sessions thereafter (Table 1). Blood flow rates of 300-350 ml/min have achieved *Kt*/*V* values of 2.7–3.0 during 8-hour nocturnal sessions and 1.5–1.8 during 5-hour daytime sessions. Two kidney transplants (1976 and 1979) failed from hyperacute rejection, though histologic documentation is unavailable [4]; dialysis continued uninterrupted thereafter.

**Table 1:** Timeline of key clinical events over the course of treatment.

Date	Event
1954	Patient born; family history consistent with hereditary nephritis (Alport syndrome)
1975 (15 March/1 May)	Cimino AV fistula created (left forearm); chronic home HD initiated
1975–1983	Home HD performed with assistance of patient’s father (Fresenius 2008 system)
1976/1979	Two kidney transplantations; both failed due to hyperacute rejection
1983–2011	In-center HD, 3×/week, 5-hour sessions
2011–June 2024	Nocturnal HD, 3×/week, 8-hour sessions
December 2022	Gastrointestinal bleeding from grade II–III esophageal varices
2023	Computed tomography: no cirrhosis or splenomegaly
2024	Progressive immobility; visual impairment from bilateral cataracts
August 2024	Vitrectomy and intracapsular lens extraction, right eye
Mid-2024–present	Resumed daytime HD sessions
November 2025	Cardiac MRI: mildly reduced left ventricular function (LVF); no pulmonary hypertension, valvular disease, or amyloidosis

Comorbidities include neurofibromatosis type 1, uremic mixed-type polyneuropathy, and lumbar vertebral fractures, none of which compromised adherence. In December 2022, he developed gastrointestinal bleeding from grade II–III esophageal varices without cirrhosis or splenomegaly on subsequent imaging. In 2024, bilateral cataracts caused progressive visual impairment, requiring vitrectomy and lens extraction. Cardiac evaluation, including magnetic resonance imaging (MRI) in November 2025, showed only mildly reduced left ventricular function without pulmonary hypertension, valvular disease, or amyloidosis. Notably, he experienced no access infections, carpal tunnel syndrome, or other classical long-term dialysis complications.

This case represents one of the longest reported durations of uninterrupted HD worldwide. Fifty years of native fistula function without revision or monitoring is, to our knowledge, unprecedented. The absence of dialysis-related amyloidosis and cardiovascular events may reflect both excellent dialysis adequacy and the comparatively favorable cardiovascular risk profile associated with Alport syndrome [1, 2]. His transplant course adds to the limited literature on kidney transplantation outcomes in Alport syndrome [4]. Extended nocturnal HD from 2011 to 2024 improved *Kt*/*V* and subjective well-being, consistent with reports linking thrice-weekly nocturnal HD to a survival benefit over conventional in-center HD [5]. His subsequent return to daytime dialysis in 2024, prompted by declining mobility and vision, did not compromise adequacy. He currently resides in a residential care home and requires only little assistance with activities of daily living; overall, he remains in good spirits and maintains a positive outlook.

Beyond the technical findings, this case underscores the psychosocial resilience required for such extraordinary treatment continuity: the patient adapted successfully across five decades of technological and medical change, from home HD performed by a family member to fully staffed in-center care. His course highlights the potential benefits of early arteriovenous fistula (AV) creation, extended and nocturnal dialysis schedules, and sustained patient engagement in achieving ultra-long-term survival on HD. As a single-patient observation, this report cannot establish generalizable predictors of longevity on HD, but it documents, in detail, the evolution of dialysis practice over half a century and offers a benchmark for vascular access durability and long-term dialysis adequacy against which future cases of exceptional survival may be compared.
